# The global prevalence of depression, anxiety, stress, and, insomnia and its changes among health professionals during COVID-19 pandemic: A rapid systematic review and meta-analysis

**DOI:** 10.1016/j.heliyon.2021.e07393

**Published:** 2021-06-26

**Authors:** Sultan Mahmud, Sorif Hossain, Abdul Muyeed, Md Mynul Islam, Md. Mohsin

**Affiliations:** a Institute of Statistical Research and Training, University of Dhaka, Bangladesh; b Department of Statistics, Jatiya Kabi Kazi Nazrul Islam University, Trishal, Mymensingh-2224, Bangladesh

**Keywords:** COVID-19, Depression, Anxiety, Stress, Insomnia, Meta-analysis, Health professionals

## Abstract

**Background:**

During the COVID-19 pandemic, the health professionals who are at the frontline of this crisis have been facing extreme psychological disorders. This research aims to provide an overall scenario of the prevalence of depression, anxiety, stress, as well as insomnia and to inspect the changes in these prevalence over time by analyzing the existing evidence during this COVID-19 pandemic.

**Methods:**

A systematic search was performed on March 30, 2021, in PubMed, MEDLINE, Google Scholar databases, and Web of Science. To assess the heterogeneity, *Q-*test, $I^{2}$ statistics, and Meta regression and to search for the publication bias, Eggers's test and funnel plot were used. The random-effect model and subgroup analysis were performed due to the significant heterogeneity.

**Results:**

Among eighty-three eligible studies in the final synthesis, 69 studies (n = 144649) assessed the depression prevalence of 37.12% (95% CI: 31.80–42.43), 75 studies (n = 147435) reported the anxiety prevalence of 41.42% (95% CI: 36.17–46.54), 41 studies (n = 82783) assessed the stress prevalence of 44.86% (95% CI: 36.98–52.74), 21 studies (n = 33370) enunciated the insomnia prevalence of 43.76% (95% CI: 35.83–51.68). The severity of the mental health problems among health professionals increased over the time during January 2020 to September 2020.

**Limitations:**

A significant level of heterogeneity was found among psychological measurement tools and across studies.

**Conclusions:**

Therefore, it is an emergency to develop psychological interventions that can protect the mental health of vulnerable groups like health professionals.

## Highlights

• The Coronavirus (COVID-19) pandemic has caused unprecedented risks to mental health among health workers globally.
• The prevalence of mental health disorder namely depression, anxiety, stress, and insomnia were significantly increased over the time from January 2020 to September 2020 among the teams working closer to infected patients.
• Comparatively higher prevalence of depression, anxiety, stress, and insomnia were observed from previous studies on medical health professionals during COVID-19 pandemic.
• It is an emergency to develop psychological interventions that can protect the mental health of vulnerable groups like health professionals.

## 1 Introduction

Since the first case was observed in December 2019 in Wuhan, the novel coronavirus has become a global health threat [1] by spreading among all the countries of the world [2, 3]. Afterward, the disease caused by the novel coronavirus (2019-nCoV) was named COVID-19 and declared a pandemic by the WHO [4, 5]. Though in recent times, many regions of the world have been experiencing eventually less transmission [1], the psychological crisis may insist for months or years [6]. Several studies showed that COVID-19 is highly associated with mental health problems in the general population and medical health workers. The health care workers are considered as the front liner for privation and controlling the current pandemic and taking care of a large number of infected and suspected patients [7]. “The COVID-19 pandemic has reminded us of the vital role health workers play to relieve suffering and save lives,” said Dr. Tedros Adhanom Ghebreyesus, WHO Director-General. Despite having a high risk of getting infected by the COVID-19, health workers are constantly providing services with huge work pressure and negative emotional stress. This breakneck and dubious environment and traumatic situations have an impact not only on the health professionals, but also, their family members, friends, and colleagues. This psychological crisis among health personals is instigated by fear, mental distress, anxiety, depression, and insomnia [8, 9, 10]. During this pandemic, health workers lost their lives not only from the virus itself but also from cardiac arrest and other conditions caused by being overworked, depressed, and having insomnia. Thus, as the most vulnerable group of being affected in direct dealing with coronavirus, the physical and mental health of health professionals deserve extensive attention [9].

During the pandemic, plenty of studies have been carried out to investigate the prevalence of clinical symptoms namely emotional distress, depression, stress, mood swings, irritability, insomnia, attention deficit hyperactivity disorder, post-traumatic stress in different countries or territories. However, the prevalence estimates differ remarkably from place to place and time to time, and from population to population. As the available studies showed different findings all over the world for the impact of the COVID-19 pandemic on mental health, a meta-analysis will be reasonable to understand the overall picture of the prevalence of depression, anxiety, stress, and insomnia among health care workers.

Though few systematic reviews and meta-analyses had been done on this, all the studies included very few articles and were mostly collected only from China. A meta-analysis and systematic review of 19 studies [11] considered studies that were conducted only in China. A systematic review with 29 studies (21 studies from China and 8 studies from 6 other countries) observed the prevalence of depression anxiety and stress among frontline healthcare workers [12]. Another meta-analysis and systematic review [13] of 12 studies was conducted that included studies only from China (11 studies) and Singapore (1 study). Mental health was considered in a meta-analysis of 12 studies among Chinese health workers [14]. Furthermore, several systematic reviews and meta-analyses [15, 16, 17, 18] were also conducted to inspect the prevalence of clinical symptoms such as depression, anxiety, sleep disturbance, and stress among the general population. However, not a single of the studies did inspect the variation over time in the prevalence of depression, anxiety, stress, and insomnia. Hence, we decided to conduct an extensive systematic review and meta-analysis by considering a large number of studies on the prevalence of those mental health problems among health care workers during the COVID-19 pandemic. Our vision also is to sketch the changes in the prevalence of depression, anxiety, stress, and insomnia among health professionals during the pandemic.

## 2 Methods

This systematic review was conducted and recoded according to the PRISMA (Preferred Reporting Items for Systematic Reviews and Meta-Analyses) Statement [19].

### 2.1 Search strategy and selection criteria

This comprehensive systematic review of published research papers on the prevalence of depression, anxiety, stress, and insomnia among health professionals during the COVID-19 pandemic was conducted by searching the following databases: PubMed, MEDLINE, Google Scholar, and Web of Science. The searches were performed on March 30, 2021, using the keywords “Coronavirus”, “COVID-19”, “SARS-Cov-2”, “Mental health problem”, “Mental health condition”, “Depression”, “Anxiety”, “Stress”, “Insomnia”, “Health care workers”, “Health professionals” and all possible combination of keywords were used.

(((((((((((((Coronavirus [Title]) OR (COVID-19 [Title])) OR (SARS-cov- [Title])) AND (2019-ncov [Title])) AND (Mental health problem [Title])) OR (Mental health condition [Title])) AND (Depression [Title])) OR (Anxiety [Title])) OR (Stress [Title])) OR (Insomnia [Title])) AND (health professionals [Title])) OR (Health care worker [Title]))))))))))))

For maximizing the comprehensiveness of the study, the list of article references was manually reviewed. However, we considered only those studies published in English, and no time and geographical constraints were imposed in the searching procedure. The preprints published on medrxiv, PsyArXiv, bioRxiv, and SSRN were also considered due to the rapid transmission of the information during the COVID-19 pandemic. All the identified research articles were transformed into EndNote (Version X8.1) reference management software.

### 2.2 Inclusion/exclusion criteria

Studies that satisfied the following criteria were included in the review: 1) the prevalence of stress/anxiety/depression/insomnia among healthcare workers during the COVID-19 pandemic, 2) written in English, 3) observational study (non-interventional study), 4) studies with available full text. The exclusion criteria were considered as follows: 1) unrelated topic, 2) studies without sufficient data such as where study site, study period, and sample size were not reported clearly, 3) more than one sources, 4) interventional studies/case reports/Editorial comments/Letters to Editor, etc. 5) articles that do not contain at least one of the information of depression, anxiety, stress, and insomnia, and 6) articles for that the full text is not available.

### 2.3 Study selection

**Figure 1:**
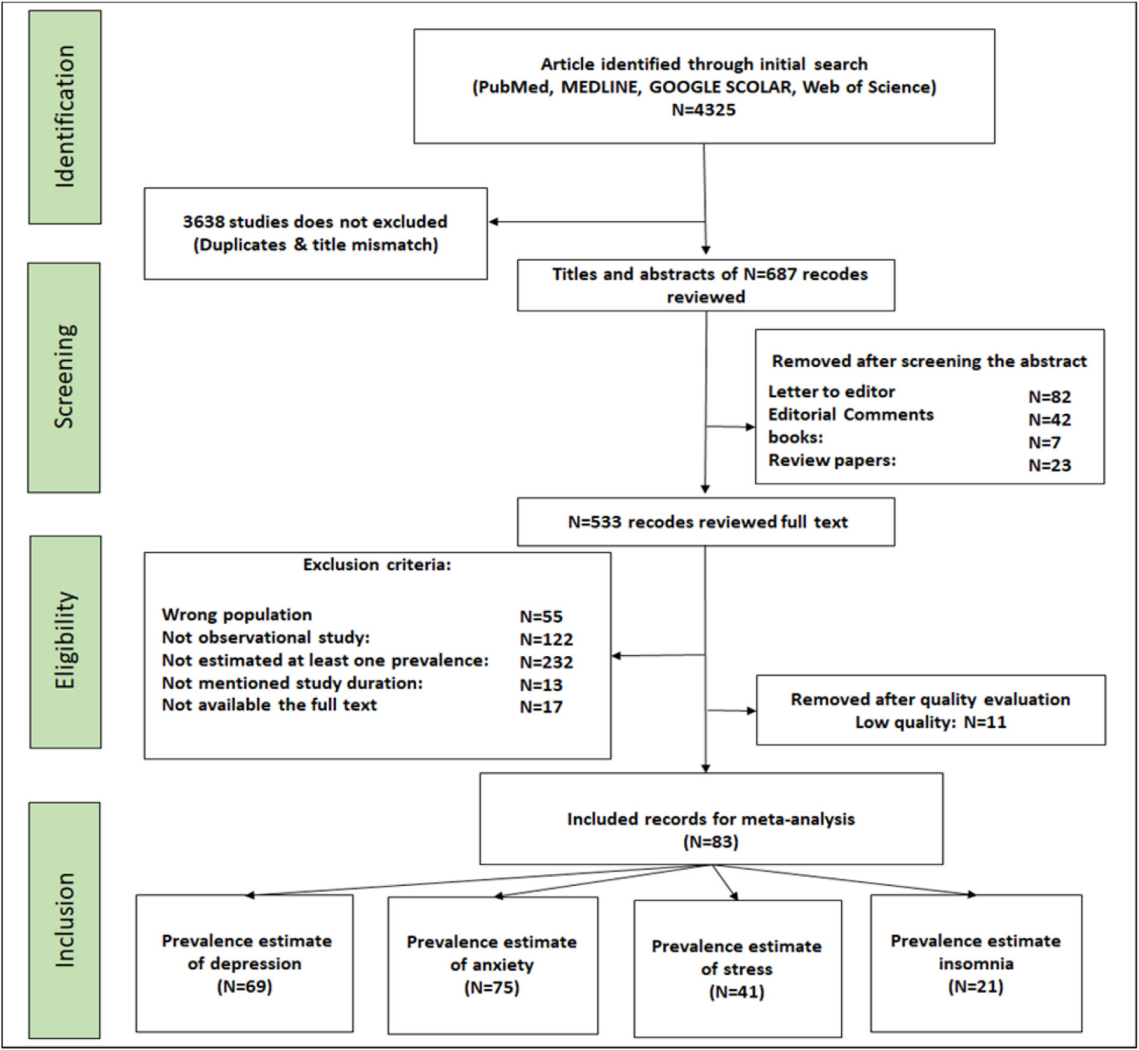
Flowchart describing the search strategy and inclusion/exclusion of studies by following the Preferred Reporting Items for Systematic Reviews and Meta-Analyses (PRISMA 2009) guidelines.

The study selection processes are sketched as follows (Figure 1). Initially, we identified articles according to PRISMA guidelines and listed them in Endnote (version X.8). In the next stage, the titles and abstracts were screened and the unrelated articles to the topics or duplicates records were removed. Then, in the eligibility checking stage, we examined the full text and skipped some articles because of not having the relevant information. In the last or final stage, all articles selected in the previous stages were reviewed or checked for quality assessment and the low-quality studies were removed.

### 2.4 Quality evaluation

The quality evaluation was carried out using the Strengthening the Reporting of Observational Studies in Epidemiology (STROBE) statements by two independent reviewers (AM and SH) along with the help of a third reviewer (SM) in case of any disagreement. The STROBE checklists contain a total of 32 scales that can be organized into six general sections/scales such as title, abstract, introduction, methods, results, and discussion. These checklists are commonly used to evaluate the quality of the observational studies in a sense of methodological aspect including sampling and data collection approaches, study population, study design, statistical methods, and so on. The maximum possible score for STROBE checklists is 32, studies with a quality evaluation score less than 14 using STROBE checklists were excluded by considering 14 as a cut-off point [15, 16].

### 2.5 Screening and extraction

Two investigators (SM and SH) independently screened and extracted data from selected articles using standardized abstraction sheets following a predefined protocol. The following information was extracted from eligible studies: first author and published year; population size; study period; response rate; geographical region (study location); ration of respondents (Doctors, nurses, others); the percentage of male in the sample; psychological assessment tools (GAD-7, PHQ-9, ISI, DASS-21, etc.) and cut-point for each of the tools; and prevalence measure of depression, anxiety, stress, and insomnia.

### 2.6 Statistical analysis

In this systematic review and meta-analysis, the data analysis was done by using Stata 16 statistical software version [20]. The Q-test and $I^{2}$ statistics [21] with a 5% level of significance were applied to assess the heterogeneity of the included studies. The random-effects model was used in case of significant heterogeneity. The Eggers’ test and funnel plot were used to search for publication bias. The subgroup analysis also was conducted by the study duration and assessment tools. To inspect the trend in psychological morbidities we inspected the prevalence of depression, anxiety, stress, and insomnia after every three months. In the article searching steps, we included studies that were conducted between January 2020 to September 2020. Then we classified all the studies into three groups: January 2020 to March 2020, April 2020 to June 2020, July 2020 to September 2020. We also used Meta-regression [22, 23] to observe the association between study duration and prevalence of psychological morbidity (prevalence of depression, anxiety, stress, and insomnia).

### 2.7 Ethical standards disclosure

This study depended on the examination of previous relevant research studies on the mental health of health professionals during COVID-19. The systematic review was done by considering all standard formats of doing systematic review given https://link.springer.com/chapter/10.1007/978-3-658-27602-7_3. This study only applies to medical health professionals and only considering mental health issues. This study removed all types of biases and provide maximum transparency of research findings to the audience.

## 3 Results

In this systematic review and meta-analysis, the prevalence of depression, anxiety, stress, and insomnia were assessed during the COVID-19 pandemic among health professionals. The PRISMA guidelines were followed for collecting and reviewing the articles in this focus. In the initial search, we identified 4325 articles, then 3638 articles were removed that were unrelated to the topics or duplicate records after screening the titles and abstracts. Next, the full texts were examined for the remaining 533 articles and 436 records were skipped because of not having the relevant information. The rest of the articles were reviewed or checked for quality and 11 studies were excluded due to the low quality. After the quality evaluation, 86 studies entered into the meta-synthesis. The article searching and selecting processes were demonstrated in the Flow Diagram following the PRISMA statements (Figure 1).

### 3.1 Study design

The studies selected for the final analysis are observational studies with a cross-sectional study design.

### 3.2 Study characteristics

Table 1 abbreviates the characteristics of the selected studies [2,8,24,25,26,27,28,29,30,31,32,33,34,35,36,37,38,39,40,41,42,43,44,45,46,47,48,49,50,51,52,53,54,55,56,57,58,59,60,61,62,63,64,65,66,67,68,69,70,71,72,73,74,75,76,77,78,79,80,81,82,83,84,85,86,87,88,89,90,91,92,93,94,95,96,97,98,99,100,101,102,103,104], 30 of which were from China, 6 from Italy, 6 from India, 4 from the United States, 3 from each of Pakistan and Iran, 2 from each of the country: Canada, Bangladesh, Egypt, Oman, Spain, Turkey, United Kingdom, 1 study from each of the country: Australia, Croatia, Finland, Germany, Ghana, Greek, Jordan, Korea, Lebanon, Nepal, Philippines, Poland, Singapore, South Korea, Vietnam, 1 jointly from Singapore and India, and another 1 jointly from Egypt and Saudi Arabia. The characteristics of the included articles were described by Study duration, Study type, First authors and year of publication, Study population, Response rate (%), Region, Physicians (%), Nurses (%), Other health professionals (%), Male (%), Assessment tools with Cut-off pint. A total of 83 studies along with an average of 78.01% response rate and with 160477 participants were considered for the final analysis. The median number of respondents was 668 (range 57–44992) with a median response rate of 90.7% (range 15–100). Among the total participants, on an average, 37.51% (median = 30.28%, range: 0%–100%) were physicians, 50.16% (median = 46.75%, range: 0%–100%) were nurses, and 30.33% (median = 29.66%, range: 0%–82%) were other health professionals. Also, among all the health professionals who participated, 29.97% were male. The average STROBE score was 21.54 with a median score of 22 (range: 15–28).

**Table 1:** Characteristics of the included studies.

Author	Duration	Study type	Study population	Response rate	Region	Physicians %	Nurses %	Other %	Male %	Assessment tools with cut-off points	STROBE score	Depression % (95% CI)	Anxiety % (95% CI)	Stress % (95% CI)	Insomnia % (95% CI)
Guo et al. (2020) [45]	February, 2020	Cross-sectional	11118	N.A.	China	30.28	53.07	16.65	25.2	SDS ≥ 50 SAS ≥ 50	26	13.4 (12.77–14.03)	4.98 (4.58–5.38)	N.A.	N.A.
Huang and Zhao (2020) [49]	Feb 3-Feb 17, 2020	Cross-sectional	7236	85.3	China	N.A.	N.A.	N.A.	45.4	CES-D ≥ 28 GAD-7 ≥ 9 PSQI > 7	22	20.1 (19.18–21.02)	35.1 (34–36.2)	N.A.	18.2 (17.31–19.09)
Lai et al. (2020) [54]	Jan 29-Feb 3, 2020	Cross-sectional	1257	68.7	China	39.2	60.8	0	23.3	PHQ-9 ≥ 5 GAD-7 ≥ 5 IES-R ≥ 9 ISI ≥ 8	24	50.4 (47.64–53.16)	44.6 (41.85–47.35)	71.5 (69–74)	34 (31.38–36.62)
Liu et al. (2020a) [61]	Feb 10-Feb 20, 2020	Cross-sectional	512	85.4	China	N.A.	N.A.	N.A.	15.4	SAS ≥ 50	23	N.A.	12.5 (9.64–15.36)	N.A.	N.A.
Lu et al. (2020) [64]	Feb 25–26, 2020	Cross-sectional	2299	94.88	China	88.8	N.A.	11.2	22.4	HAM-D ≥ 7 HAM-A ≥ 7	17	11.8 (10.48–13.12)	22.6 (20.89–24.31)	N.A.	N.A.
Qi et al. (2020) [75]	February, 2020	Cross-sectional	1306	93.6	China	N.A.	N.A.	N.A.	19.6	AIS >6	20	N.A.	N.A.	N.A.	45.5 (42.8–48.2)
Zhang et al. (2020a) [2]	Jan 29-Feb 3, 2020	Cross-sectional	1563	N.A.	China	29	62.9	8.1	13.5	PHQ-9 ≥ 5 GAD-7 ≥ 5 IES-R ≥ 9 ISI ≥ 8	22	50.7 (48.22–53.18)	44.7 (42.24–47.16)	73.4 (71.21–75.59)	36.1 (33.72–38.48)
Zhang et al. (2020c) [8]	Feb 19-Mar 6, 2020	Cross-sectional	2182	N.A.	China	31.2	11.3	57.5	35.8	PHQ-2 ≥ 3 GAD-2 ≥ 3 ISI >8	22	10.6 (9.31–11.89)	10.4 (9.12–11.68)	N.A.	33.9 (31.91–35.89)
Zhu et al. (2020a) [97]	Feb 8–10, 2020	Cross-sectional	5062	77.1	China	19.8	67.5	12.7	15	PHQ-9 ≥ 10 GAD-7 ≥ 8 IES-R ≥ 33	22	13.45 (12.51–14.39)	24.06 (22.88–25.24)	29.8 (28.54–31.06)	N.A.
Chew et al. (2020) [34]	Feb 19-April 17, 2020	Cross-sectional	906	90.6	Singapore and India	29.6	39.2	31.2	35.7	DASS-21 > 9 DASS-21 > 7 DASS-21 > 14	16	10.6 (8.6–12.6)	15.7 (13.33–18.07)	5.2 (3.75–6.65)	N.A.
Mrklas et al. (2020) [69]	Mar 23-May 4, 2020	Cross-sectional	44992	19.4	Canada	N.A.	N.A.	N.A.	13.8	PHQ-9 ≥ 10 GAD-7 ≥ 10 PSS ≥ 14	22	44 (43.54–44.46)	47.47 (47.01–47.93)	85.6 (85.28–85.92)	N.A.
Xing et al. (2020) [93]	February 7 to February 21, 2020	Cross-sectional	309	100	China	12.3	88.7	N.A.	2.6	SDS ≥ 53 SAS ≥ 50	23	56 (50.47–61.53)	28.5 (23.47–33.53)	N.A.	N.A.
Huang et al. (2020) [101]	Feb 7–9, 2020	Cross-sectional	364	96.55	China	N.A.	N.A.	N.A.	41.2	SAS ≥ 50	21	N.A.	23.4 (19.05–27.75)	N.A.	N.A.
Pan et al.(2020) [102]	Feb 7–21, 2020	Cross-sectional	194	97	China	21.6	76.3	2.1	18.6	PHQ-9 ≥ 5 GAD-7 ≥ 5	22	37.6 (30.78–44.42)	32.5 (25.91–39.09)	N.A.	N.A.
AraçI & Dönmezdil (2020) [85]	13-Mar-20	Cross-sectional	210	94.28	Turkey	32.32	35.85	31.83	60.61	HADS> 11 HADS> 11	25	31.31 (25.04–37.58)	39.39 (32.78–46)	N.A.	N.A.
Alshekaili et al. (2020) [27]	8–17 Apr, 2020	Cross-sectional	1139	N.A.	Oman	33.7	39.5	26.8	20	DASS-21 ≥ 10 DASS-21 ≥ 8 DASS-21 ≥ 16 ISI ≥ 14	21	32.3 (29.58–35.02)	34.1 (31.35–36.85)	23.8 (21.33–26.27)	18.5 (16.24–20.76)
Khanal et al. (2020) [53]	Apr 26- May 12, 2020	Cross-sectional	475	N.A.	Nepal	33.9	35.2	30.9	47.4	HADS> 7 HADS> 7 ISI> 7	26	37.5 (33.15–41.85)	41.9 (37.46–46.34)	N.A.	33.9 (29.64–38.16)
Elkholy et al. (2020) [24]	Apr–May, 2020	Cross-sectional	502	N.A.	Egypt	60	16.1	23.9	50	PHQ-9 ≥ 5 GAD-7 ≥ 5 PSS ≥ 9 ISI ≥ 8	24	77.2 (73.53–80.87)	76.4 (72.69–80.11)	80.9 (77.46–84.34)	67.7 (63.61–71.79)
Smith et al. (2020) [84]	Apr 07 - May 13, 2020	Cross-sectional	7298	82.04	Canada	N.A.	N.A.	N.A.	9	PHQ-2 ≥ 3 GAD-2 ≥ 3	24	42.3 (41.17–43.43)	54.8 (53.66–55.94)	N.A.	N.A.
Liang et al. (2020) [60]	Feb 14- Mar 29, 2020	Cross-sectional	899	81.43	China	24.91	45.16	29.93	18.69	PHQ-9 ≥ 5 GAD-7 ≥ 5 ISI≥ 8	20	82.53 (80.05–85.01)	61.4 (58.22–64.58)	N.A.	43.04 (39.8–46.28)
Prasad et al. (2020) [74]	Apr 14–25, 2020	Cross-sectional	347	N.A.	United States	N.A.	71.5	28.5	9.2	PHQ-2 ≥ 3 GAD-7 ≥ 5 IES≥ 9	25	22.8 (18.39–27.21)	69.5 (64.66–74.34)	84.2 (80.36–88.04)	N.A.
Liu et al. (2020b) [62]	Feb 17 - Feb 23, 2020	Cross-sectional	2031	N.A.	China	42.25	57.75	N.A.	14.48	DASS-21 ≥ 3 DASS-21 ≥ 8 DASS-21 ≥ 15	20	14.81 (13.27–16.35)	18.3 (16.62–19.98)	9.98 (8.68–11.28)	N.A.
Evanoff et al. (2020) [40]	Apr 17- May 1, 2020	Cross-sectional	5550	34.3	United States	19.45	N.A.	80.55	21.4	DASS-21	21	15.29 (14.34–16.24)	13 (12.12–13.88)	N.A.	N.A.
Wańkowicz et al. (2020) [91]	3 May 2020 to 17 May 2020	Cross-sectional	441	N.A.	Poland	N.A.	N.A.	N.A.	47.84	PHQ-9 > 4 GAD-7 > 4 ISI > 8	23	70.74 (66.49–74.99)	64.39 (59.92–68.86)	N.A.	58.04 (53.43–62.65)
Que et al. (2020) [50]	16–23 Feb, 2020	Cross-sectional	2285	N.A.	China	37.67	9.1	53.23	30.94	PHQ-9 ≥ 5 GAD-7 ≥ 5 ISI ≥ 15	23	89 (87.72–90.28)	92.07 (90.96–93.18)	N.A.	57.5 (55.47–59.53)
Luceño-Moreno et al. (2020) [65]	1 to 30 of April, 2020	Cross-sectional	1422	92.39	Spanish	10	34.2	55.8	13.6	HADS ≥ 7 HADS ≥ 7 IES-R ≥ 20	23	51.3 (48.7–53.9)	79.3 (77.19–81.41)	83 (81.05–84.95)	N.A.
Sandesh et al. (2020) [81]	20-May	Cross-sectional	112	N.A.	Pakistan	N.A.	N.A.	N.A.	57.1	DASS-21	15	71.32 (62.94–79.7)	85.71 (79.23–92.19)	N.A.	N.A.
Giusti et al. (2020) [42]	April 16- May 11 2020	Cross-sectional	330	41.25	Italy	42.2	26	31.8	37.4	DASS-21 > 9 DASS-21 > 7 DASS-21 > 14	18	26.8 (22.02–31.58)	31.3 (26.3–36.3)	34.3 (29.18–39.42)	N.A.
Chatterjee et al. (2020) [33]	April 20- May 20 2020	Cross-sectional	308	81.16	India	40	32.9	27.1	43.6	ISI > 7	28	N.A.	N.A.	N.A.	47.9 (42.32–53.48)
Zhu et al. (2020b) [98]	February 1- February 29, 2020	Cross-sectional	165	N.A.	China	47.9	52.2	N.A.	17	SDS > 50 SAS > 50	25	45.6 (38–53.2)	11.4 (7.97–14.83)	N.A.	N.A.
Alnazly et al. (2021) [26]	August 16th to 23rd, 2020.	Cross-sectional	365	100	Jordan	10	63	27	44.4	DASS-21 > 9 DASS-21 > 7 DASS-21 > 14	27	80 (75.9–84.1)	85 (81.15–88.85)	75 (70.33–79.67)	N.A.
Ni at al. (2021) [71]	4-Mar-20	Cross-sectional	57	100	China	83	17	N.A.	17	SDS > 53 SAS > 50	15	42 (29.19–54.81)	9 (5.91–12.09)	N.A.	N.A.
Du et al. (2020) [38]	March 19-April 7, 2020	Cross-sectional	403	N.A.	China	39.21	54.84	5.96	27.27	DASS-21 > 9 DASS-21 > 7 DASS-21 > 14	24	17 (13.33–20.67)	32 (26.97–37.03)	88 (84.49–91.51)	N.A.
Hong et al. (2020) [48]	February 8 to 14, 2020	Cross-sectional	4692	N.A.	China	N.A.	100	N.A.	3.1	PHQ-9 > 10 GAD-7 > 10	20	9.4 (8.56–10.24)	8.1 (5.16–11.04)	N.A.	N.A.
Gorini et al. (2020) [43]	April 1st to May 1st 2020.	Cross-sectional	650	84.4	Italy	27	32.9	40.1	67.5	PHQ-9 > 3 GAD-7 > 9	18	22.8 (19.57–26.03)	29.6 (24.67–34.53)	44.9 (39.53–50.27)	N.A.
Lee et al. (2020) [56]	June 22 to July 8, 2020	Cross-sectional	406	N.A.	Korea	20	55.7	24.3	28.3	PHQ-9 ≥ 10 GAD-7 ≥ 5 ISI≥8	19	14.3 (10.89–17.71)	39.4 (34.13–44.67)	52 (46.61–57.39)	36.2 (30.83–41.57)
Ning et. al (2020) [72]	20-Feb	Cross-sectional	612	N.A.	China	51.8	48.2	0	27.1	SDS ≥ 53 SAS ≥ 50	22	25 (21.57–28.43)	16.3 (12.31–20.29)	N.A.	N.A.
Ahn et al. (2020) [25]	20–30 April 2020,	Cross-sectional	1783	N.A.	South Korea	16.4	54.2	29.4	23.9	PHQ-9 ≥ 10 GAD-7 ≥ 5	18	10 (8.61–11.39)	10 (6.76–13.24)	N.A.	N.A.
Tan et al. (2020) [31]	May 29 -June 24, 2020	Cross-sectional	3075	27.2	Singapore	14.9	45.3	39.8	25.8	HADS > 7 HADS > 7	23	31.8 (30.15–33.45)	40.7 (35.4–46)	N.A.	N.A.
Conti et al. (2020) [35]	March 30- May 03, 2020	Cross-sectional	933	76.3	Italy	24	42.3	33.7	23.5	PHQ-9 ≥5 GAD-7 ≥5 PHQ-15 ≥ 5	20	57.9 (54.73–61.07)	65.2 (60.06–70.34)	55 (49.63–60.37)	N.A.
Wang et al. (2020) [90]	2nd -3rd February 2020	Cross-sectional	1045	80.1	China	14.3	74	11.7	14.2	HADS > 7 ISI ≥ 8	26	39.4 (36.44–42.36)	47.8 (42.41–53.19)	N.A.	49.9 (44.32–55.48)
Lasalvia et al. (2021) [55]	21 April-6 May 2020	Cross-sectional	2195	36.9	Italy	13.9	35.7	50.4	24.7	PHQ-9 ≥ 10 SAS ≥ 36 IES-R ≥ 24	20	26.6 (24.75–28.45)	50.1 (44.71–55.49)	53.8 (48.42–59.18)	N.A.
Zheng et al. (2021) [96]	March 6, 2020, to March 9, 2020	Cross-sectional	617	N.A.	China	N.A.	100	N.A.	8.5	DASS-21 > 9 DASS-21 > 7 DASS-21 > 14	26	15.4 (12.55–18.25)	32.6 (27.54–37.66)	18 (13.85–22.15)	N.A.
Arafa et al. (2021) [30]	14th and 24th of April 2020	Cross-sectional	426	N.A.	Egypt and Saudi Arabia	48.4	24.2	27.4	50.2	DASS-21 > 9 DASS-21 > 7 DASS-21 > 14	25	69 (64.61–73.39)	58.9 (53.59–64.21)	55.9 (50.54–61.26)	N.A.
Lenzo et al. (2021) [58]	April 27 to May 4, 2020	Cross-sectional	214	N.A.	Italy	19.6	80.4	0	39	DASS-21 > 9 DASS-21 > 7 DASS-21 > 14	25	23 (17.36–28.64)	18 (13.85–22.15)	24 (19.39–28.61)	N.A.
Sunil et al. (2021) [86]	June 1, 2020, to July 4, 2020	Cross-sectional	313	N.A.	India	100	N.A.	N.A.	35.5	PHQ-4>2 PHQ-4>2 PSS>13 ISI>7	24	47 (41.47–52.53)	47 (41.61–52.39)	54 (48.62–59.38)	31.9 (26.69–37.11)
Hennein et al. (2021) [47]	20-May	Cross-sectional	1092	96.5	US	31.2	19	49.8	28	PHQ-9 ≥ 10 GAD-7 ≥ 10 PTSD ≥ 3	22	13.9 (11.85–15.95)	15.6 (11.68–19.52)	22.8 (18.27–27.33)	N.A.
Mathur et al. (2020) [67]	20-Jun	Cross-sectional	200	N.A.	India	87	13	N.A.	69	DASS-21 ≥ 10 DASS-21 ≥ 8 DASS-21 ≥ 15	22	17 (11.79–22.21)	19.5 (15.23–23.77)	9.5 (6.34–12.66)	N.A.
Liu et al. (2021) [63]	24 February to 9 March, 2020	Cross-sectional	1090	N.A.	China	40.6	59.4	N.A.	19.8	PHQ-9 ≥ 15 GAD-7 ≥ 15 PSS-10 ≥ 15	22	18.4 (16.1–20.7)	13.3 (9.64–16.96)	48.07 (42.68–53.46)	N.A.
Tasnim et al. (2020) [87]	July and August 2020	Cross-sectional	803	97	Bangladesh	68	10	22	50.7	HADS ≥ 8 HADS ≥ 8	23	39.5 (36.12–42.88)	69.5 (64.53–74.47)	N.A.	N.A.
Greene et al. (2021) [44]	27 May to 23 July, 2020	Cross-sectional	1194	49	UK	3.85	42.21	53.94	7.04	PHQ-9 ≥ 10 GAD-7 ≥ 8	16	47 (44.17–49.83)	47 (41.61–52.39)	N.A.	N.A.
Ranjan et al. (2021) [76]	23 April to 24 May, 2020	Cross-sectional	373	N.A.	India	19.3	42.1	38.6	47.7	DASS-21	15	39.1 (34.15–44.05)	53.6 (48.22–58.98)	62.5 (57.28–67.72)	N.A.
Si et al. (2020) [83]	23 February to 5 March, 2020	Cross-sectional	863	76	China	43.7	24.4	31.9	29.3	DASS-21 ≥ 10 DASS-21 ≥ 10 IES-6 ≥ 10	23	13.6 (11.31–15.89)	13.9 (10.17–17.63)	8.6 (5.58–11.62)	N.A.
Khalaf et al. (2020) [52]	March to May, 2020	Cross-sectional	170	N.A.	Egypt	100	N.A.	N.A.	38.8	DASS-21 ≥ 10 DASS-21 ≥ 8 DASS-21 ≥ 15	24	63 (55.74–70.26)	77.6 (73.1–82.1)	72 (67.16–76.84)	N.A.
Tu et al. (2020) [89]	7 February to 25 February, 2020	Cross-sectional	100	100	China	N.A.	100	N.A.	N.A.	PHQ-9 ≥ 15 GAD-7 ≥ 4	23	46 (36.23–55.77)	40 (34.71–45.29)	N.A.	60 (54.53–65.47)
Nguyen et al. (2021) [70]	22 April to 12 May, 2020	Cross-sectional	761	98.3	Vietnam	59.9	N.A.	40.1	41.8	IES-R ≥ 24	17	N.A.	N.A.	34.3 (29.18–39.42)	N.A.
Ferini-Strambi et al. (2020) [41]	Feb, 2020–May, 2020	Cross-sectional	7236	N.A.	Italy	30	70	N.A.	30	PSQI>5	20	N.A.	N.A.	N.A.	35 (29.67–40.33)
Mattila et al. (2021) [39]	Mar 20-June 20, 2020	Cross-sectional	1995	19.13	Finland	6	66	28	13	GAD-7 ≥ 5	24	N.A.	45 (39.63–50.37)	N.A.	N.A.
Chen et al. (2020) [104]	Jan 23-Mar 22, 2020	Cross-sectional	1493	93.31	China	28.27	N.A.	N.A.	55.39	SDS>53 PSS-10 > 20	19	3.79 (2.82–4.76)	N.A.	71.56 (66.69–76.43)	N.A.
Li et al. (2020) [77]	Jan–Feb 2020	Cross-sectional	176	100	China	N.A.	100	N.A.	22.7	HAM-A>7	18	N.A.	77.3 (72.78–81.82)	N.A.	N.A.
An et al. (2020) [94]	Mar 15-Mar 20, 2020	Cross-sectional	1103	100	China	N.A.	100	N.A.	N.A.	PHQ-9 ≥ 5	21	43.61 (40.68–46.54)	N.A.	N.A.	N.A.
Holton et al. (2020) [82]	May 15- June 10, 2020	Cross-sectional	668	15	Australia	20.65	58.53	20.82	N.A.	DASS-21 ≥ 5 DASS-21 ≥ 5 DASS-21 ≥ 5	19	23 (19.81–26.19)	29 (24.1–33.9)	25 (20.33–29.67)	N.A.
Ofori et al. (2021) [28]	July 11- August 12, 2020	Cross-sectional	272	90.7	Ghana	N.A.	N.A.	N.A.	48.73	DASS-21 ≥ 10 DASS-21 ≥ 8 DASS-21 ≥ 15	22	21.1 (16.25–25.95)	27.8 (22.97–32.63)	8.2 (5.24–11.16)	N.A.
Bizri et al. (2021) [68]	Apr–May, 2020	Cross-sectional	150	18.75	Lebanon	27.7	37.3	35	44	IES-R ≥ 33	23	N.A.	N.A.	33 (27.93–38.07)	N.A.
Wilson et al. (2020) [103]	Apr 10-Apr 25, 2020	Cross-sectional	433	80.83	India	84.3	15.7	N.A.	53.4	PHQ-9 ≥ 10 GAD-7 ≥ 5 PSS ≥ 9	18	49.4 (44.69–54.11)	66.7 (61.62–71.78)	82.6 (78.51–86.69)	N.A.
Badahdah et al. (2020) [100]	Apr 1- Apr 14, 2020	Cross-sectional	509	N.A.	Oman	38.1	61.9	N.A.	19.7	GAD-7 ≥ 5 PSS ≥ 24	22	N.A.	74.1 (69.37–78.83)	43.6 (38.25–48.95)	N.A.
Wasim et al. (2020) [88]	May 20-June 3rd, 2020	Cross-sectional	356	N.A.	India	60.11	21.91	17.98	48.03	DASS-21 ≥ 10 DASS-21 ≥ 8 DASS-21 ≥ 15 ISI ≥ 8	19	62.35 (57.32–67.38)	64.76 (59.61–69.91)	55.33 (49.97–60.69)	53.37 (47.8–58.94)
Lamb et al. (2021) [36]	Apr–June, 2020	Cross-sectional	4378	94	UK	12.8	26.7	60.5	24.6	PHQ-9 ≥ 10 GAD-7 ≥ 10 PTSD-8 ≥ 14	18	27.3 (25.98–28.62)	23.2 (18.65–27.75)	30.2 (25.25–35.15)	N.A.
Blekas et al. (2020) [29]	Apr 10 -Apr 13, 2020	Cross-sectional	2427	96	Greek	N.A.	N.A.	N.A.	21.9	PHQ-9>6 PDI>10 PTSD-8>9 AIS>10	19	27.23 (25.46–29)	41.33 (36.02–46.64)	34.13 (29.01–39.25)	15.34 (11.32–19.36)
Hasan et al. (2020) [46]	Apr 21-May 10, 2020	Cross-sectional	442	93.21	Bangladesh	100	N.A.	N.A.	44.2	HADS ≥ 8 HADS ≥ 8	25	67.72 (63.36–72.08)	48.5 (43.11–53.89)	N.A.	N.A.
Labrague et al. (2020) [59]	Apr 25-May 25, 2020	Cross-sectional	350	92.85	Philippines	N.A.	100	N.A.	N.A.	PSSQ ≥ 5	21	N.A.	37.8 (32.57–43.03)	N.A.	N.A.
Salman et al. (2020) [79]	Apr 15-May 20, 2020	Cross-sectional	398	N.A.	Pakistan	52	N.A.	48	N.A.	PHQ-9 ≥ 10 GAD-7 ≥ 10	19	21.9 (17.84–25.96)	21.4 (16.97–25.83)	N.A.	N.A.
Hassannia et al. (2020) [57]	Apr 6-Apr 15, 2020	Cross-sectional	2200	93	Iran	6.2	5.1	N.A.	32.5	HADS>8 HADS>8	22	42.3 (40.24–44.36)	65.6 (60.47–70.73)	N.A.	N.A.
Hassamal et al. (2021) [80]	April 30 to May 22, 2020	Cross-sectional	3500	36	US	12.2	39.8	48	22.2	PHQ-9 ≥ 10 GAD-7 ≥ 8	24	21 (19.65–22.35)	33 (27.93–38.07)	N.A.	N.A.
Kaveh et al. (2020) [66]	Mar 25- Mar 31, 2020	Cross-sectional	1072	96.82	Iran	N.A.	49.5	N.A.	12.4	BAI ≥ 8	18	N.A.	39.6 (34.32–44.88)	N.A.	N.A.
Saleem et al. (2020) [99]	4-Apr-20	Cross-sectional	404	N.A.	Pakistan	30.7	18.8	50.5	34.9	FCV-19S ≥ 10	20	N.A.	79.7 (75.36–84.04)	N.A.	N.A.
Aziznejadroshan et al. (2020) [73]	May–June, 2020	Cross-sectional	224	N.A.	Iran	N.A.	100	N.A.	25.4	DASS-21 ≥ 10 DASS-21 ≥ 8 DASS-21 ≥ 10	20	43 (36.52–49.48)	54 (48.62–59.38)	17.4 (13.31–21.49)	N.A.
Zhang et al. (2020b) [95]	Jun 6- Jun 13, 2020	Cross-sectional	721	90	China	25.66	69.03	5.31	14.95	HADS ≥ 8 HADS ≥ 8 ISI ≥ 8	25	82.09 (79.29–84.89)	88.88 (85.49–92.27)	N.A.	95.52 (93.21–97.83)
Cai et al. (2020) [32]	Feb 11- Feb 26, 2020	Cross-sectional	1173	N.A.	China	98.6	N.A.	1.4	30.2	PHQ-9>10 BAI>15 ISI ≥ 9	26	14.3 (12.3–16.3)	15.7 (11.77–19.63)	N.A.	47.8 (42.22–53.38)
Elbay et al. (2020) [78]	Mar 10-Mar 15, 2020	Cross-sectional	442	100	Turkey	17.2	N.A.	82.8	43.21	N.A.	25	64.7 (60.24–69.16)	51.6 (46.21–56.99)	41.2 (35.89–46.51)	N.A.
Mira et al. (2020) [51]	Mar 18-May 17, 2020	Cross-sectional	685	33.33	Spain	28.6	39	32.4	N.A.	EASE ≥ 10	18	N.A.	N.A.	27.4 (22.59–32.21)	N.A.
Salopek-Žiha et al. (2020) [37]	Mar 26 -Apr 6 2020	Cross-sectional	124	100	Croatia	22	78	0	N.A.	N.A.	20	11 (5.49–16.51)	17 (12.95–21.05)	10 (6.76–13.24)	N.A.
Weibelzahl et al. (2021) [92]	May 22-July 22, 2020	Cross-sectional	300	98.67	Germany	N.A.	N.A.	N.A.	18.91	ISR ≥ 10 ISR ≥ 10	21	82 (77.65–86.35)	47.77 (42.38–53.16)	N.A.	N.A.

N.A., Not available; CES-D, Center for Epidemiologic Studies Depression Scale; DASS-21, The Depression, Anxiety and Stress Scale; GAD-7, Generalized Anxiety Disorder 7-item; PHQ-9, Patient Health Questionnaire; SAS, The Zung Self-Rating Anxiety Scale; SDS, The Zung Self-Rating Depression Scale; HAM-A, Hamilton Anxiety Scale Rating; HAM-D, Hamilton Rating Depression Scale; IES-R, Impact of Events Scale – Revised; PSQI, The Pittsburgh Sleep Quality Index; ISI, The Insomnia Severity Index; HADS, Hospital Anxiety and Depression Scale; IES, Impact of Event Scale; PSS, The Perceived Stress Scale; PTSD-8, The Post-traumatic Stress Disorder-8 Scale; EASE, The Examination of Anomalous Self-Experience Scale; FCV-19S, The Fear of COVID-19 Scale.

### 3.3 Statistical heterogeneity and publication bias

**Figure 2:**
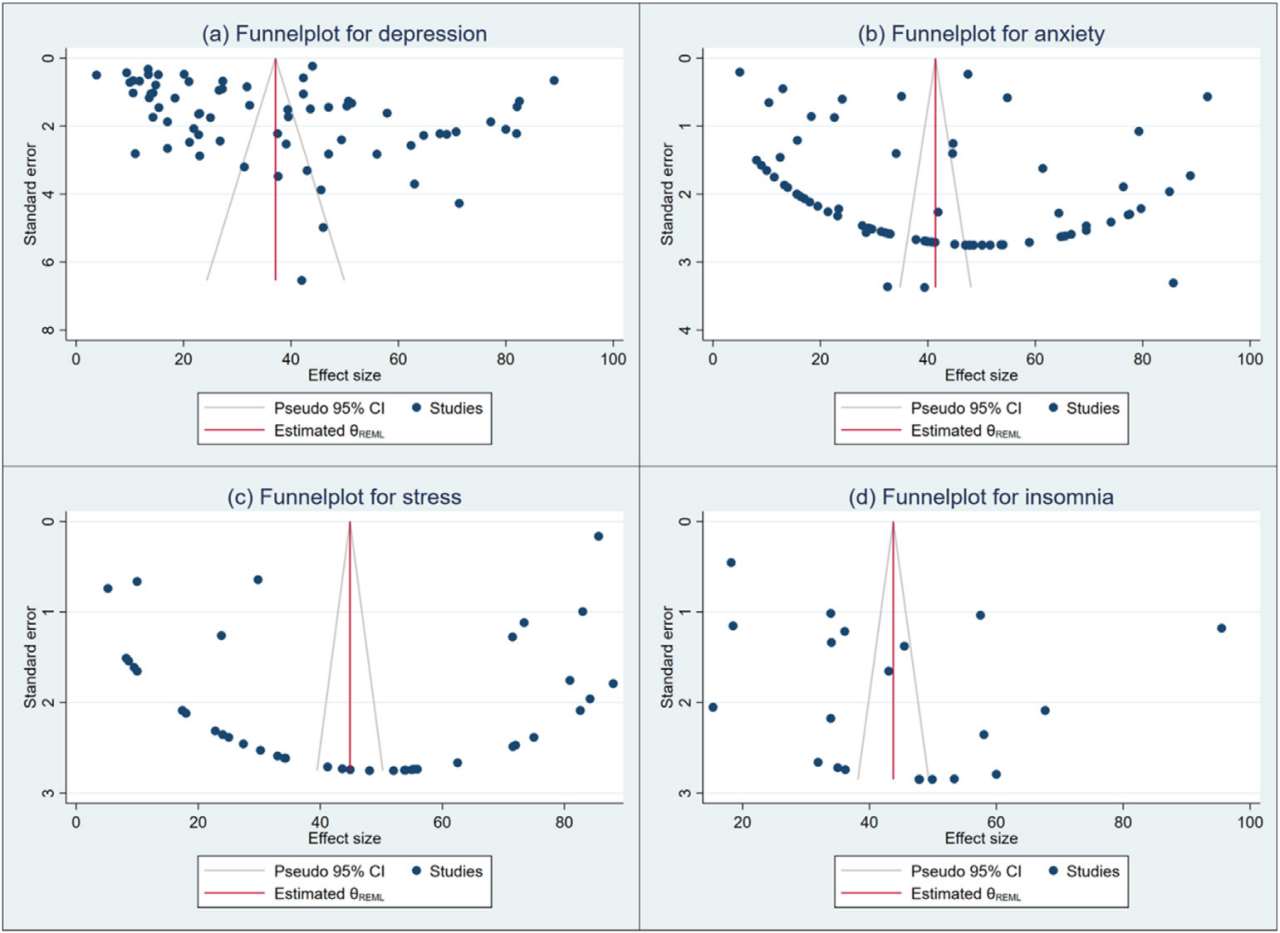
Funnel plot of result of the prevalence of depression (a), anxiety (b), stress (c), and insomnia (d) among health professionals.

Two statistical tools *Q-*test and $I^{2}$ (%) were used to investigate the heterogeneity of the included studies. Here, we obtained for depression (*Q* (68) = 34675.82, *P* < 0.05) $I^{2}$ = 99.84%, anxiety (*Q* (74) = 47974.95, *P* < 0.05) $I^{2}$ = 99.79%, stress (*Q* (40) = 40900.83, *P* < 0.05) $I^{2}$ = 99.78%, and, insomnia (*Q* (20) = 5390.03, *P* < 0.05) $I^{2}$ = 99.41%. We prefer to use a random-effects model for analyzing the outputs due to higher heterogeneity among the studies. We also performed the Meta-regression to search for the source of heterogeneity. The results of individual variable meta-regression models show (Table 2) that the high heterogeneity of the included studies for depression and anxiety was associated with study duration and sample size. The high heterogeneity among the included studies for stress was associated with measurement tools and for insomnia with study duration. The Funnel plot and Eggers's test indices were used to investigate the publication bias for selected studies. The results indicate that there is no significant publication bias for stress (*Z* = 0.33, *P* = 0.74) (Figure 2c), and insomnia (*Z* = 0.67, *P* = 0.50) (Figure 2d). However, a significant publication bias was found for depression (*Z* = 2.77, *P* = 0.005) (Figure 2a), and anxiety (*Z* = 2.22, *P* = 0.02) (Figure 2b).

**Table 2:** Result of individual variable meta-regression models for each stratified meta-analysis.

Variables	Label	Prevalence (95% CI)	Coefficient (95% CI)	P-value
**Depression**				
Study duration	January to March, 2020	32.5 (23.86–41.15)	(Ref)	
	April to June, 2020	39.62 (32.01–47.24)	7.13 (-4.58–18.84)	0.03
	July to September, 2020	46.88 (12.8–80.96)	14.39 (-12.26–41.05)	0.02
Response rate	Less than 60%	29.43 (21.75–37.12	(Ref)	
	Greater than 60%	40.11 (32.05–48.17)	10.67 (-5.97–27.31)	0.21
Sample size	Greater than 600	30.96 (24.28–37.63)	(Ref)	
	Less than 600	45.24 (37.43–53.05)	14.28 (4.03–24.54)	0.01
Measurement tools	CES-D	20.1 (19.18–21.02)	(Ref)	
	DASS-21	34.83 (24.81–44.84)	14.72 (-30.69–60.13)	0.53
	HADS	47.02 (35.74–58.3)	26.91 (-19.86–73.68)	0.26
	PHQ	38.11 (29.03–47.19)	15.5 (-31.93–62.93)	0.52
	SDS	30.63 (14.27–46.99)	18.01 (-27.23–63.25)	0.44
	Other^#^	35.62 (15.28–55.96)	10.59 (-37.39–58.57)	0.67
**Anxiety**				
Study duration	January to March, 2020	30.68 (22.97–38.39)	(Ref)	
	April to June, 2020	48.57 (40.68–56.47)	17.88 (5.9–29.87)	0.00
	July to September, 2020	60.79 (27.31–94.27)	30.11 (1.75–58.47)	0.04
Response rate	Less than 60%	37.3 (29.34–45.26)	(Ref)	
	Greater than 60%	42.34 (34.97–49.72)	4.98 (-10.2–20.16)	0.52
Sample size	Greater than 600	36.99 (29.84–44.15)	(Ref)	
	Less than 600	46.24 (38.71–53.76)	9.24 (-1.13–19.62)	0.08
Measurement tools	BAI	27.58 (4.16–51)	(Ref)	
	DASS-21	39.62 (29.41–49.84)	-19.97 (-67.69–27.74)	0.41
	GAD	43.66 (35.27–52.05)	3.72 (-44.42–51.85)	0.88
	HADS	58.06 (46.02–70.1)	29.2 (-36.39–94.79)	0.38
	HAMA	49.91 (-3.7–103.51)	(empty)	
	SAS	19.39 (9.4–29.37)	(empty)	
	Other^@^	43.91 (25.94–61.88)	-11.4 (-61.63–38.83)	0.66
**Stress**				
Study duration	January to March, 2020	38.14 (21.41–54.87)	(Ref)	
	April to June, 2020	46.31 (36.47–56.15)	8.17 (-10.84–27.17)	0.40
	July to September, 2020	41.58 (-23.89–107.04)	3.37 (-34.62–41.37)	0.86
Response rate	Less than 60%	43.26 (24.64–61.88)	(Ref)	
	Greater than 60%	41.63 (28.72–54.54)	-1.62 (-26.08–22.85)	0.90
Sample size	Greater than 600	40.77 (30.12–51.42)	(Ref)	
	Less than 600	49.17 (37.53–60.81)	8.4 (-7.35–24.15)	0.30
Measurement tools	DASS-21	35.25 (23.03–47.46)	(Ref)	
	IES	65.95 (49.26–82.64)	30.71 (7.92–53.5)	0.01
	PSS	69.46 (52.68–86.23)	5.63 (-13.48–24.74)	0.56
	PTSD	26.43 (19.18–33.68)	34.17 (9.7–58.64)	0.01
	Other^$^	40.84 (30.04–51.65)	-8.74 (-44.85–27.37)	0.64
**Insomnia**				
Study duration	January to March, 2020	42.46 (34.68–50.23)	(Ref)	
	April to June, 2020	48.79 (30.38–67.19)	6.25 (-12.28–24.78)	0.05
	July to September, 2020	-	(empty)	
Response rate	Less than 60%	-	(Ref)	
	Greater than 60%	45.47 (29.87–61.07)	(empty)	
Sample size	Greater than 600	40.79 (29.4–52.18)	(Ref)	
	Less than 600	48.64 (39.32–57.96)	7.85 (-8.54–24.24)	0.35
Measurement tools	AIS	30.46 (0.9–60.01)	(Ref)	
	ISI	46.58 (37.7–55.46)	44.643 (-43.421–132.707)	0.32
	PSQI	26.39 (9.93–42.85)	85.516 (-149.536–320.568)	0.47

Ref, reference group.

Other ^#^ includes ISR and HAMD.

Other^@^ includes FCV-19S, ISR, PDI, PHQ-4, PSSQ.

Other^$^ includes EASE, PHQ-15, SAVE-9.

### 3.4 The pooled prevalence

**Figure 3:**
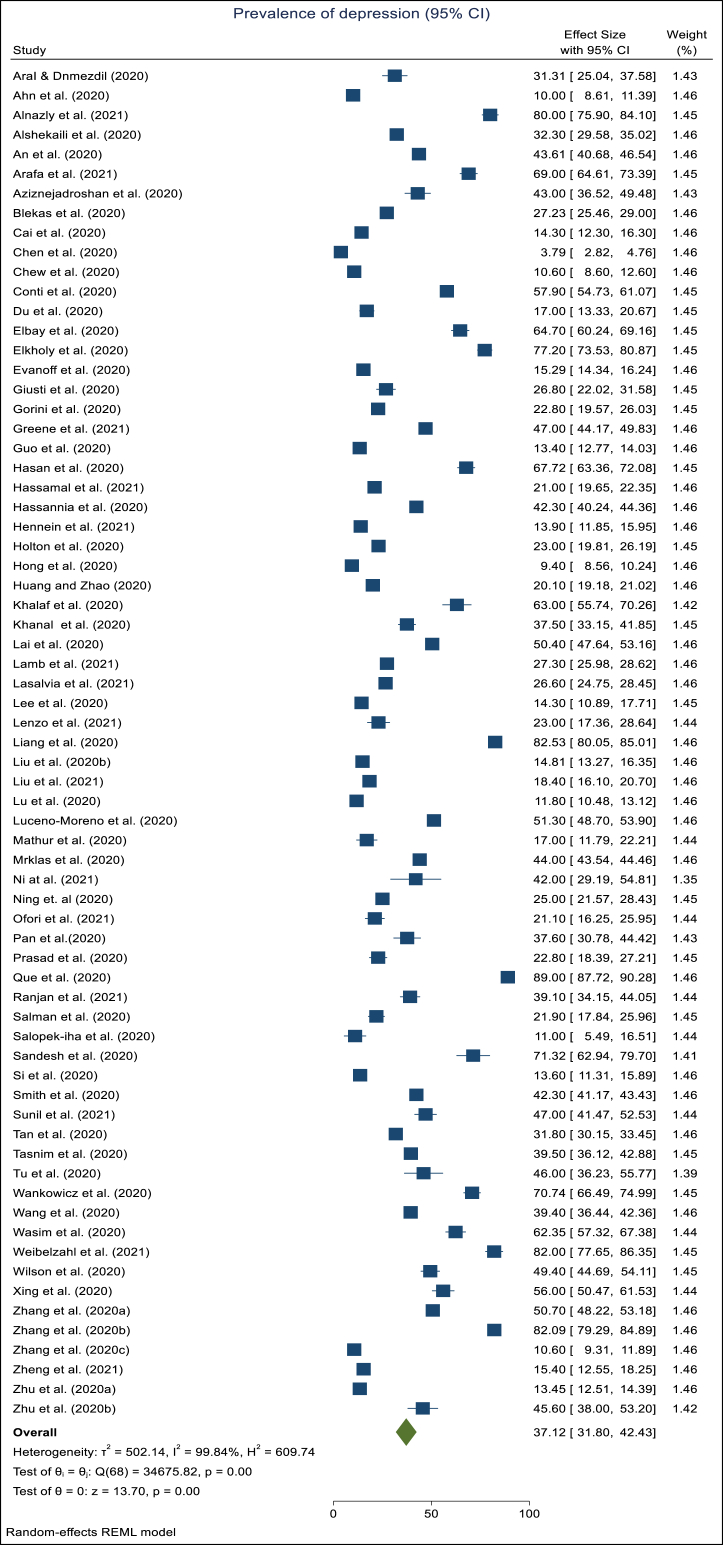
Forest plot showing the meta-analyses of the pooled prevalence of depression among health professionals during the pandemic.

**Figure 4:**
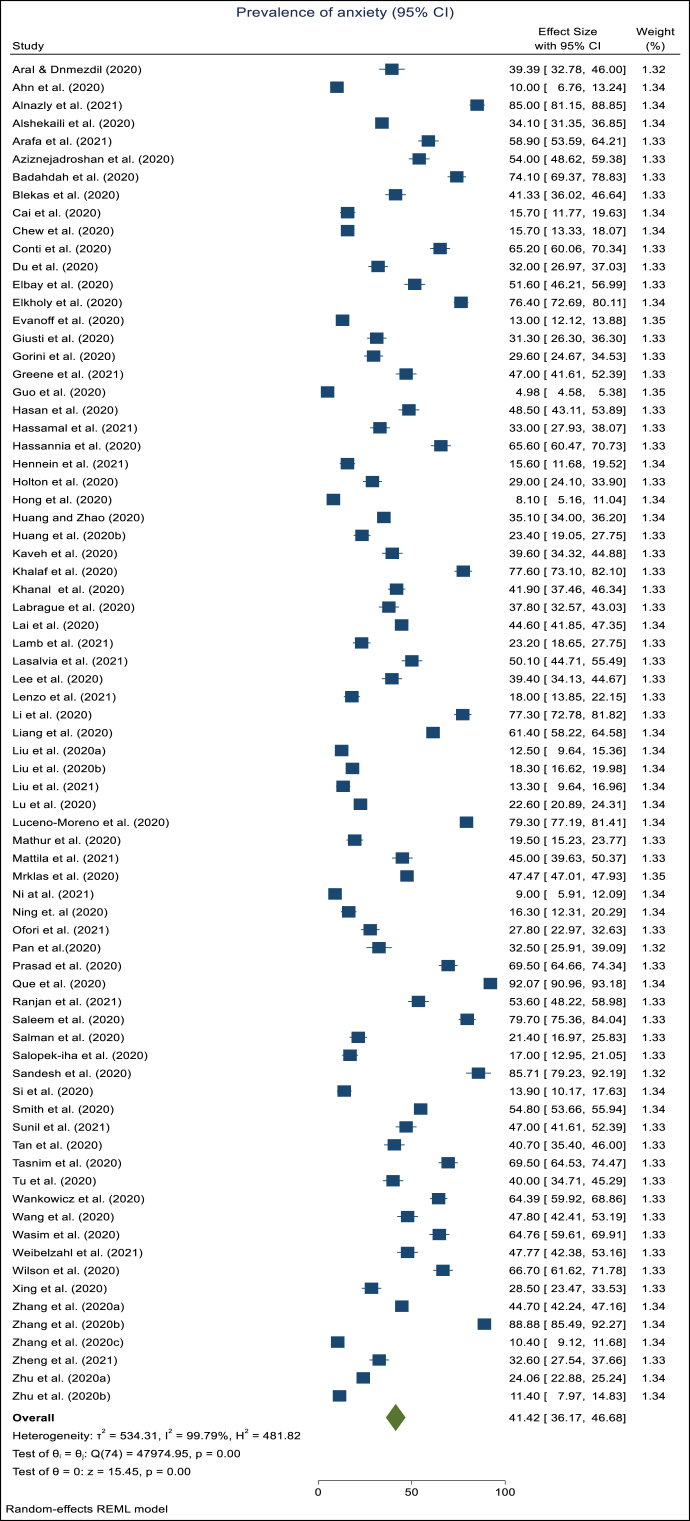
Forest plot showing the meta-analyses of the pooled prevalence of anxiety among health professionals during the pandemic.

**Figure 5:**
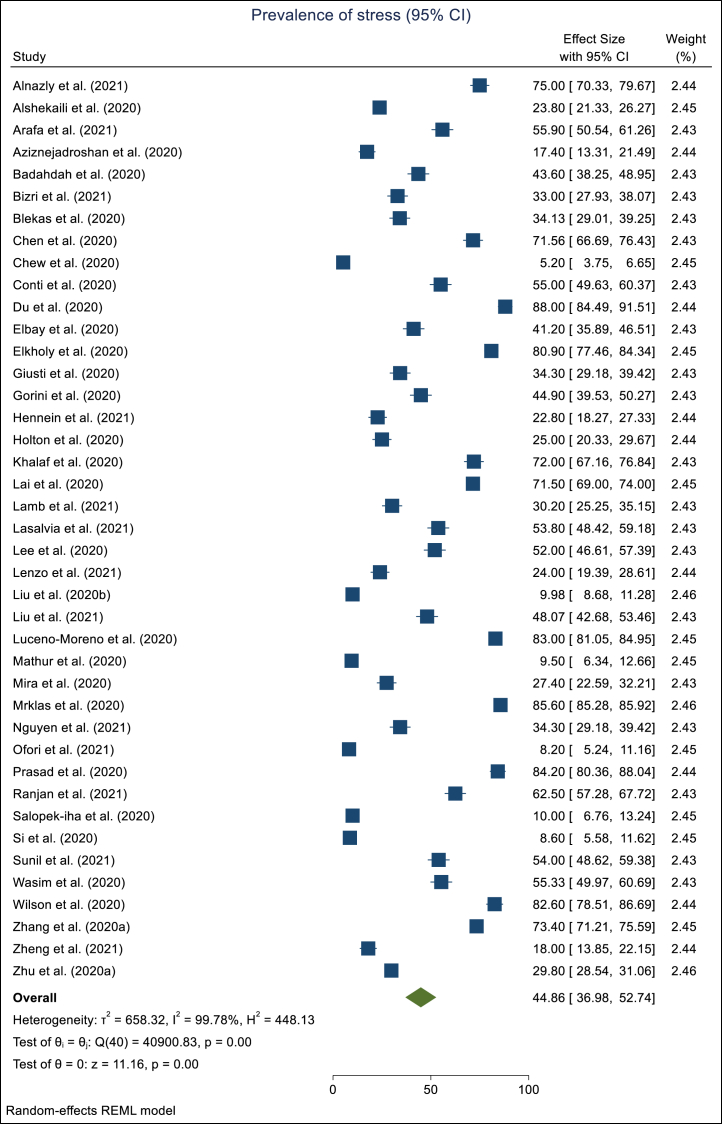
Forest plot showing the meta-analyses of the pooled prevalence of stress among health professionals during the pandemic.

**Figure 6:**
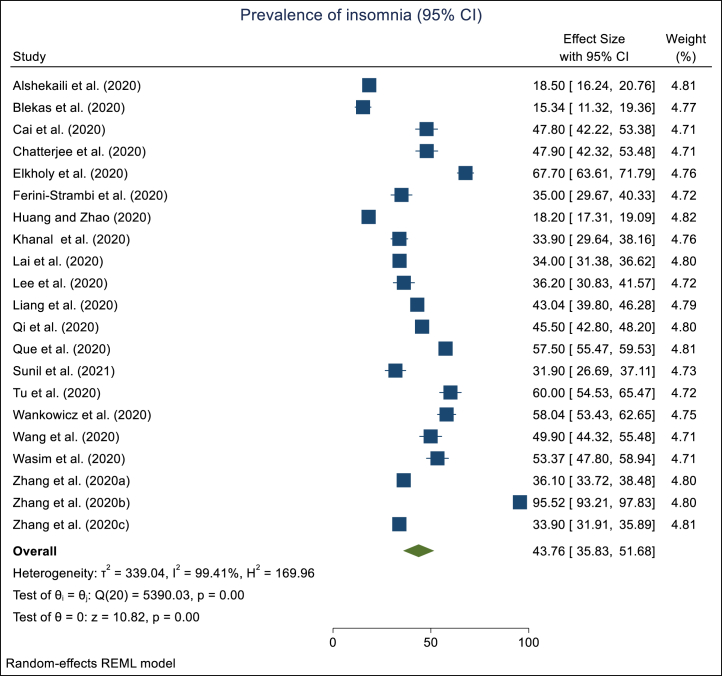
Forest plot showing the meta-analyses of the pooled prevalence of insomnia among health professionals during the pandemic.

Depression was reported in 69 studies (n = 144649) out of 83 studies. The pooled prevalence was estimated as 37.12% (95% CI: 31.80–42.43) (Figure 3). The between study heterogeneity was found statistically significant ($I^{2}$= 99.84%, P-value = 0.00) (Figure 3). Anxiety was mentioned in 75 studies (n = 147435) from 83 studies. The pooled prevalence of anxiety was assessed as 41.42% (95% CI: 36.17–46.54) (Figure 4) with a significant between study heterogeneity ($I^{2}$= 99.79%, P-value = 0.00) (Figure 4). Stress was reported in 41 studies (n = 82783) with a pooled prevalence of 44.86% (95% CI: 36.98–52.74) (Figure 5). There was significant serious between-study heterogeneity ($I^{2}$= 99.78%, P-value = 0.00) (Figure 5). Similarly, the pooled prevalence of insomnia was estimated from 21 studies (n = 33370) as 43.76% (95% CI: 35.83–51.68) (Figure 6). A severe ($I^{2}$= 99.41%, P-value = 0.00) between study heterogeneity was also found (Figure 6).

### 3.5 Subgroup analysis

The subgroup analysis of depression, anxiety, stress, and insomnia was conducted by study duration, assessment tools, and country.

#### 3.5.1 Study duration subgroups

Based on the availability of data, all the studies were further classified into three three-month groups: January 2020 to March 2020, April 2020 to June 2020, July 2020 to September 2020. However, 13 studies [34, 35, 37, 38, 39, 41, 44, 51, 52, 56, 69, 86, 92] out of the 83 were not classified into any of those groups due to inconvenient study duration. Finally, 70 studies were included in the subgroup analysis based on study duration. Among them, 59 studies have the estimates of depression prevalence, 64 studies have the estimates of anxiety prevalence, 32 studies have the estimates of stress prevalence and 18 studies have estimates of insomnia prevalence.

**Figure 7:**
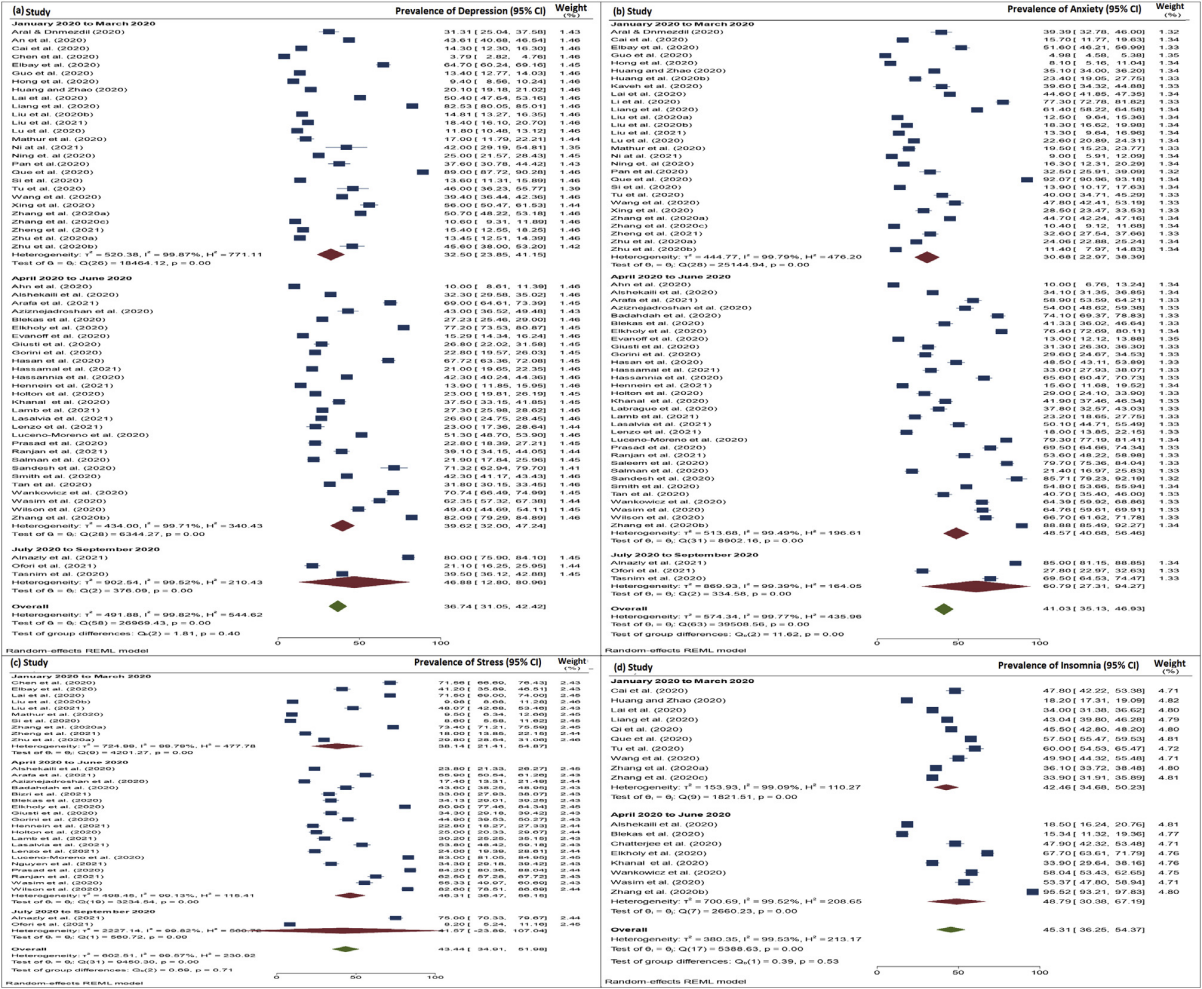
Forest plot showing changes of the pooled prevalence of depression (a), anxiety (b), stress (c), and insomnia (d) between study duration January 2020 to September 2020, among health professionals.

**Figure 8:**
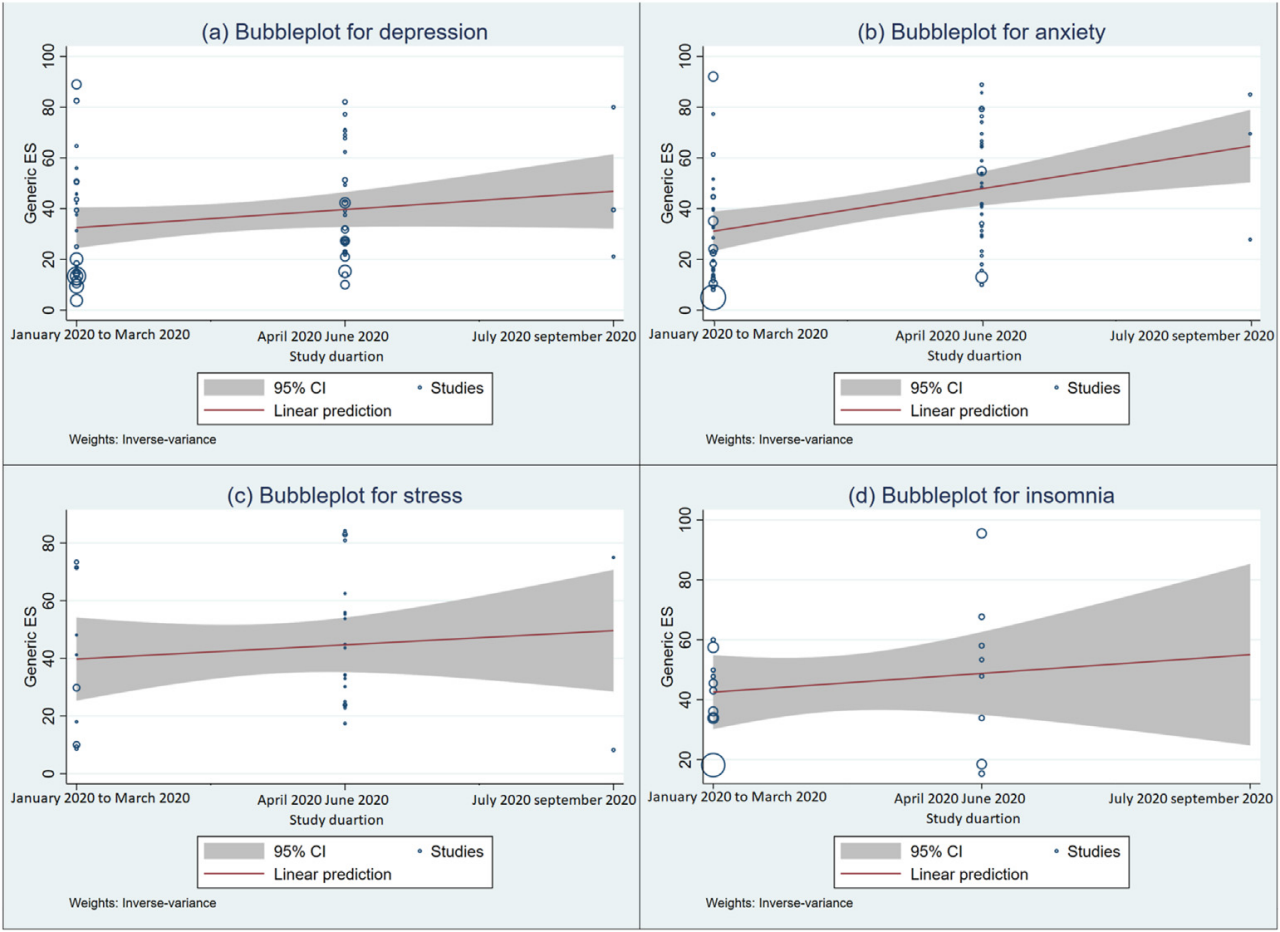
Bubble plots from meta-regression showing the changes in the prevalence of depression (a), anxiety (b), stress (c), and insomnia (d) with study duration.

Twenty-seven studies were conducted between January 2020 to March 2020, with a pooled depression prevalence of 32.50%. Twenty-eight studies took place between April 2020 to June 2020 with a pooled depression prevalence of 39.62%. Three studies took place between July 2020 to September 2020 with a pooled depression prevalence of 46.88% (Please see Figure 7(a)). With a pooled anxiety prevalence of 30.68%, twenty-nine studies occurred between January 2020 to March 2020, thirty-two studies with a pooled anxiety prevalence of 48.57% were conducted between April 2020 to June 2020, and three studies were conducted between July 2020 to September 2020 with a pooled anxiety prevalence of 60.79% (Please see Figure 7(b)). Ten studies were conducted between January to March 2020 with a pooled stress prevalence of 38.14%, and four studies were carried out during April 2020 and June 2020, with a pooled stress prevalence of 46.31%, and two studies were carried out from July 2020 to September 2020 with a pooled stress prevalence of 41.57% (Please see Figure 7(c)). With a pooled insomnia prevalence of 42.46%, fourteen studies were done between January 2020 to March 2020, eight studies with a pooled insomnia prevalence of 48.79% were conducted from April 2020 to June 2020, and no study has been conducted from July 2020 to September 2020 with an estimate of insomnia prevalence (Please see Figure 7(d)). An upward trend in the prevalence of depression and anxiety was observed from January 2020 to September 2020 which was also satisfied by the Meta-regression findings (Figure 8 and Table 2).

#### 3.5.2 Country subgroups

Table 3 narrated the results of subgroup analysis of depression, anxiety, stress, and insomnia prevalence by country. Two studies [30, 34] covered populations from two different countries, and those were not included in this subgroup analysis. The results show the pooled prevalence of depression was the highest in Germany, (82.00% with 95% CI: 77.65–86.35) and the lowest in South Korea (10.00% with 95% CI: 8.61–11.93), anxiety was the highest in Jordan (85.00% with 95% CI: 81.33–88.67), and the lowest in South Korea, (10% with 95% CI: 6.92–13.08), stress was the highest in Canada (85.60% with 95% CI: 85.28–85.92) [69] and the lowest in Ghana (8.20% with 95% CI: 5.38–11.02), insomnia was the highest in Egypt (67.70% with 95% CI: 64.59–70.81) and the lowest in Greece (15.34% with 95% CI: 11.64–19.04).

**Table 3:** Subgroup analysis of depression, anxiety, stress, and, insomnia prevalence by country.

Region	Depression % (95% CI) ($I^{2}$, Q (df.) P-value)	Anxiety % (95% CI) ($I^{2}$, Q (df.) P-value)	Stress % (95% CI) ($I^{2}$, Q (df.) P-value)	Insomnia % (95% CI) ($I^{2}$, Q (df.) P-value)
Australia	23.00 (19.81–26.19)∗	29.00(24.34−33.66)∗	25.00(20.55−29.45)∗	-
China	33.23 (23.80–42.67) (Q (25) = 19950.90, $I^{2}$ = 99.89, p-value<0.001)	31.89 (22.78–41.00) (Q (26) = 26562.16, $I^{2}=99.85$, p-value < 0.001)	46.53 (26.43–66.63) (Q (8) = 4954.56, $I^{2}=99.85$, p-value < 0.001)	44.50 (34.76–54.21) (Q (13) = 5197.16, $I^{2}=99.53$, p-value < 0.001)
Bangladesh	53.57 (25.92–81.23) (Q (1) = 100.53, $I^{2}=99.01$, p-value<0.001)	59.03 (38.45–79.60) (Q (1) = 34.76, $I^{2}=97.12$, p-value < 0.001)	-	-
Canada	43.23 (41.57–44.89) (Q (1) = 7.46, $I^{2}=1.25$, p-value = 0.006)	51.12 (43.93–58.30) (Q (1) = 136.58, $I^{2}=99.27$, p-value < 0.001)	85.60(85.28−85.92)∗	-
Croatia	11.00(5.49−16.51)∗	17.00(13.14−20.86)∗	10.00(6.92−13.08)∗	-
Egypt	70.46 (56.56–84.36) (Q (2) = 13.04, ${\text{I}}^{2}=91.03,$p-value = 0.001)	76.88 (74.02–79.75) (Q (1) = 0.16, $I^{2}=0,$p-value = 0.68)	76.62 (67.91–85.34) (Q (1) = 8.63, $I^{2}=88.41$, p-value = 0.003)	67.7 (64.59–70.81)∗
Finland	-	45.00(39.89−50.11)∗	-	-
Germany	82.00(77.65−86.35)∗	47.77(42.64−52.90)∗	-	-
Ghana	21.10(16.25−25.95)∗	27.80(23.20−32.40)∗	8.20(5.38−11.02)∗	-
Greek	27.23(25.46−29.00)∗	41.33(36.27−46.39)∗	34.13(29.26−39.00)∗	15.34(11.64−19.04)∗
India	42.98 (28.30–57.66) (Q (4) = 164, $I^{2}=97.61,$p-value < 0.001)	50.28 (33.55–67.01) (Q (4) = 295.14, $I^{2}=98.37$, p-value < 0.001)	52.76 (29.23–76.29) (Q (4) = 966.73, $I^{2}=99.35$, p-value < 0.001)	44.36 (31.71–57.01) (Q (2) = 39.57, $I^{2}=94.77$, p-value < 0.001)
Iran	42.36 (40.40–44.33) (Q (1) = 0.04, $I^{2}=0,$ p-value = 0.84)	53.08 (38.33–67.83) (Q (2) = 53.04, $I^{2}=96.16$, p-value < 0.001)	17.40(13.51−21.30)∗	-
Italy	31.47 (18.33–44.61) (Q (4) = 334.06, $I^{2}=98.75$, p-value < 0.001)	38.81 (22.41–55.20) (Q (4) = 256.35, $I^{2}=98.39$, p-value < 0.001)	42.36 (30.74–53.97) (Q (4) = 119.98, $I^{2}=96.43$, p-value < 0.001)	35.00(30.10−39.90)∗
Jordan	80.00(75.90−84.14)∗	85.00(81.33−88.67)∗	75.00(70.55−79.45)∗	-
Korea	14.3(10.90−17.71)∗	39.40(34.38−44.42)∗	52.00(46.87−57.13)∗	36.20(31.26−41.14)∗
Lebanon		-	33.00(28.17−37.83)∗	-
Nepal	37.50(33.15−41.85)∗	41.90(37.46−46.34)∗	-	33.90(29.64−38.16)∗
Oman	32.30(29.58−35.02)∗	54.06 (14.86–93.26) (Q (1) = 220.95, $I^{2}=99.55$, p-value < 0.001)	33.57 (14.17–52.97) (Q (1) = 46.98, $I^{2}=97.87$, p-value < 0.001)	18.5(16.24−20.76)∗
Pakistan	46.47 (-1.96–94.90) (Q (1) = 108.17, $I^{2}=99.08$, p-value < 0.001)	62.23 (22.01–102.45) (Q (2) = 465.58, $I^{2}=99.53$, p-value < 0.001)	-	-
Philippines		37.80(32.82−42.78)	-	-
Poland	70.74(66.49−74.99)∗	64.39(59.92−68.86)∗	-	58.04(53.43−62.65)
Singapore	31.8(30.15−33.45)∗	40.70(35.65−45.75)∗	-	-
South Korea	10.00(8.61−11.93)∗	10.00(6.92−13.08)∗	-	-
Spain	51.30(48.70−53.90)∗	79.30(77.19−81.41)∗	55.24 (0.75–109.73) (Q (1) = 478.91, $I^{2}=99.79$, p-value < 0.001)	-
Turkey	48.08 (15.36–80.08) (Q (1) = 72.39, $I^{2}=98.62,$p-value<0.001)	45.68 (33.72–57.64) (Q (1) = 8.18, $I^{2}=87.77$, p-value = 0.004)	41.20(36.14−46.26)∗	-
United Kingdom	37.11 (17.80–56.41) (Q (1) = 152.81, $I^{2}=99.35$, p-value < 0.001)	35.06 (11.73–58.38) (Q (1) = 48.25, $I^{2}=97.93$, p-value < 0.001)	30.20(25.48−34.92)∗	-
United States	18.01 (13.91–22.11) (Q (3) = 61.64, $I^{2}=95.86$, p-value < 0.001)	32.72 (7.22–58.22) (Q (3) = 559.53, $I^{2}=99.60$, p-value < 0.001)	53.51 (-6.66–113.69) (Q (1) = 434.61, $I^{2}=99.77$, p-value < 0.001)	-
Vietnam	-	-	34.30(29.42−39.18)∗	-
Overall	37.04 (31.71–42.37) (Q (66) = 34051.22, $I^{2}=99.83$, p-value < 0.001)	41.54 (36.20–46.87) (Q (72) = 47722.64, $I^{2}=99.80$, p-value < 0.001)	45.60 (37.59–53.62) (Q (38) = 32786, $I^{2}=99.76$, p-value < 0.001)	58.04 (53.43–62.65) (Q (24) = 6102.53, $I^{2}=99.33$, p-value < 0.001)

- refers there is no studies were considered in this subgroup.

CI, Confidence Interval.

∗ refers only one studies were included in this subgroup.

#### 3.5.3 Assessment tools subgroups

Table 4 shows the highest pooled depression prevalence of 47.02% (95% CI: 35.74–58.30) for the HADS tool reported in nine studies and the lowest pooled depression prevalence of 20.10% (95% CI: 19.10–21.02) for the CES-D tool reported in only one study. The highest pooled anxiety prevalence of 58.06% (95% CI: 46.02–70.10) for the HADS tool was reported in nine studies and the lowest pooled anxiety prevalence of 19.39% (95% CI: 9.40–29.37) for the SAS tool was reported in eight studies. The highest stress and insomnia pooled prevalence of 65.95% (95% CI: 49.26–82.64) for IES and 46.58% (95% CI: 37.70–55.46) for ISI were reported in twenty-seven and sixteen studies respectively and the lowest stress and insomnia pooled prevalence of 26.43% (95% CI: 19.18–33.68) for PTSD was reported in two studies and 26.39% (95% CI: 9.93–42.84) for PSQI was reported in two studies respectively.

**Table 4:** Subgroup analysis of depression, anxiety, stress, and, insomnia prevalence by assessment tools.

Tools	Prevalence of Depression (%) (95% CI) ($I^{2}$, Q (df), P-value)	Tools	Prevalence of Anxiety (%) (95% CI) ($I^{2}$ , Q (df), P-value)	Tools	Prevalence of Stress (%) (95% CI) ($I^{2}$, Q (df), P-value)	Tools	Prevalence of Stress (%) (95% CI) ($I^{2}$, Q (df), P-value)
SDS	30.63 (14.27, 46.98) ($I^{2}$= 99.81, Q (5) = 684.88, p-value < 0.001)	SAS	19.39 (9.40, 29.37) ($I^{2}$= 99.08, Q (7) = 478.65, p-value < 0.001)	PSS	69.46 (52.68, 86.23) ($I^{2}$= 99.32, Q (4) = 374.70, p-value < 0.001)	AIS	30.46 (0.90, 60.01) ($I^{2}$= 99.33, Q (1) = 149.01, p-value < 0.001)
CES-D	$20.10\;\left(19.10,\;21.02\right){\;}^{*}$	BAI	27.58 (4.16, 51.00) ($I^{2}$= 98.03, Q (1) = 50.69, p-value < 0.001)	PTSD	26.43 (19.18, 33.68) ($I^{2}$= 78.60, Q (1) = 4.67, p-value = 0.03)	PSQI	26.39 (9.93, 42.84) ($I^{2}$= 97.31, Q (1) = 37.17, p-value < 0.001)
PHQ-9	38.11 (29.03, 47.19) ($I^{2}$= 99.59, Q (20) = 2698.05, p-value < 0.001)	GAD	43.65 (35.27, 52.05) ($I^{2}$= 99.81, Q (26) = 13807.06, p-value < 0.001)	IES	65.95 (49.26, 82.64) ($I^{2}$= 99.81, Q (26) = 13807.06, p-value < 0.001)	ISI	46.58 (37.70, 55.46) ($I^{2}$= 99.20, Q (15) = 2964.36, p-value < 0.001)
DASS-21	34.83 (24.81, 44.84) ($I^{2}$= 99.88, Q (24) = 19749.36, p-value < 0.001)	HAMA	49.91 (-3.70, 103.51) ($I^{2}$= 99.80, Q (1) = 492.23, p-value < 0.001)	DASS-21	35.24 (23.03, 47.46) ($I^{2}$= 99.69, Q (5) = 2989.42, p-value < 0.001)	-	-
HADS	47.02 (35.74, 58.30) ($I^{2}$= 99.30, Q (8) = 1107.94, p-value < 0.001)	DASS-21	39.62 (29.41, 49.84) ($I^{2}$= 99.55, Q (20) = 3420.53, p-value < 0.001)	Other	40.84 (30.04, 51.65) ($I^{2}$= 98.03, Q (9) = 647.01, p-value < 0.001)	-	-
Other	35.62 (15.28, 55.96) ($I^{2}$= 99.84, Q (6) = 2018.09, p-value < 0.001)	HADS	58.06 (46.02, 70.10) ($I^{2}$= 98.63, Q (8) = 667.96, p-value < 0.001)	-	-	-	-
-	-	Other	43.91 (25.94, 61.88) ($I^{2}$= 98.88, Q (5) = 665.18, p-value < 0.001)	-	-	-	-
Overall	37.12 (31.80, 42.43) ($I^{2}$= 99.84, Q (68) = 34675.82, p-value < 0.001)	Overall	41.42 (36.17, 46.68) ($I^{2}$= 99.79, Q (74) = 47974.95, p-value < 0.001)	Overall	44.86 (36.98, 52.74) ($I^{2}$= 99.78, Q (40) = 40900.83, p-value < 0.001)	Overall	42.95 (34.79, 51.12) ($I^{2}$= 99.44, Q (19) = 5306.39, p-value < 0.001)

∗refers only one studies were included in this subgroup

- refers there is no studies were considered in this subgroup.

CI, Confidence Interval.

## 4 Discussion

In March 2020, SARS-CoV-2 was announced as a pandemic by the WHO and more than 200 nations are at present struggling with this pandemic [105]. During the COVID-19 pandemic, the mental health of HPs faced serious problems. Death to their colleagues and threats to their lives, the fear of getting infected, the nonattendance of a strong psychological supportive system, and high workload all expanded mental health disorders among HPs [53, 106, 107]. However, only a few studies have investigated the psychiatric morbidity and mental health problems among HPs during the COVID-19 pandemic and no study has yet been conducted to find the variation of psychological status over time among health workers during the COVID-19 pandemic. In light of information extracted from the included studies, a significant proportion of the analyzed sample demonstrated a level of mental health problems among HPs.

In this meta-analysis, 83 studies were included after evaluating the overall quality of the articles with a 90.70% response rate. Among the total participants, on average, 37.51% were physicians, 50.16% were nurses, and 30.33% were other health professionals. Besides, 29.97% were males among the participated HPs (See Table 1). The pooled prevalence of depression, anxiety, stress, and insomnia was 37.12% (95% CI: 31.86–42.43) (Figure 3), 41.42% (95% CI: 36.17–46.68) (Figure 4), 44.86% (95% CI: 36.98–52.74) (Figure 5), and 43.76% (95% CI: 35.83–51.68) (Figure 6), respectively. These prevalence estimates were consistently lower in earlier studies [12, 13]. The sub-group analysis based on study duration shows that the pooled prevalence of depression and anxiety was increasing over time during the pandemic (Figures 7 and 8). The pooled prevalence of depression increased from 32.50% from January to March 2020 to 39.62% from April to June 2020. Then during July and September 2020, the prevalence estimate became 46.88% (Figures 7a and 8). Similarly, the prevalence of anxiety increased from 30.68% during January–March 2020 to 48% during April–June 2020 and then further increased to 60.79% during June–September 2020 (Figures 7b and 8). The pooled prevalence of stress during April–June 2020 rose upward to 46.31% from 38.14% during January–March 2020 but then decreased to 41.57% during June–September 2020 (Figures 7c and 8). The pooled prevalence of insomnia became 48.79% during April–June 2020 from 42.46% during January–March 2020 (see Figures 7d and 8). In our subgroup analysis, it is vivid that the prevalence of psychological morbidities varies across countries of the world. The healthcare workers in Germany were found to have the highest level of depression. HPs in Jordan faced the highest level of anxiety, in Canada the highest level of stress, and in Egypt the highest level of insomnia. The reverse scenarios (lowest estimates) were found for depression and anxiety in South Korea, for stress in Ghana, and for insomnia in Greece [28] (Table 3). The psychological tools that measured the highest pooled prevalence of depression of 47.02% using 9 studies [2, 31, 46, 53, 57, 65, 85, 87, 90], anxiety of 58.06% using 9 studies [2, 31, 46, 53, 57, 65, 85, 87, 90], stress of 69.46% using 5 studies [24, 69, 86, 100, 103], and insomnia of 46.58% using 16 studies [8, 24, 27, 32, 33, 50, 53, 54, 56, 60, 86, 88, 90, 91, 95] were HADS, HADS, PSS and ISI respectively. On the contrary, HAMD, SAS, PTSD, and PSQI tools were used to measure the lowest pooled prevalence of depression of 20.10% using one studies [49], anxiety of 19.39% using 8 studies [45, 55, 61, 71, 72, 93, 98, 101], stress of 26.43% using 2 studies [36, 47], and insomnia of 26.39% using 2 studies [41, 49] (Table 4).

Previous reports of SARS infections' acute effect on health professionals' mental health revealed that high levels of depression, anxiety, stress, fear, and insomnia were common [61, 108]. SARS virus outbreaks occurred over the last decade as an emerging infectious disease, observed first in China and afterward spread to 29 nations in 2002 [109]. A study observed that 89% of the health professionals faced psychological problems during that SARS outbreak period [54]. Another study observed the psychological impact of SARS and found the prevalence of depression, anxiety, insomnia in HPs as 74.20%, 77.4%, and 52.3%, respectively. A study [110] using the GHQ-2 questionnaire showed that around 57% of HPs suffered from psychological distress during the SARS outbreak. Similarly, in the initial stages of the COVID-19 outbreak, HPs showed higher psychological distress like depression, anxiety, stress, and insomnia which were assessed through PHQ-9, GAD-7, DASS-21, and AIS in several studies. Our findings were also consistent with the previous studies on psychological health among HPs during SARS and COVID-19 outbreak.

A worldwide pandemic can be a significant reason for fear and stress. During the SARS epidemic, HPs expressed anxiety, fear and some of them wanted to leave their job [111]. As compared to the SARS epidemic, nowadays the quick spread of information (misinformation) may cause greater public fear, depression, and trouble. HPs’ care for patients with a contagious disease (COVID-19 patients) resulted in fear, panic, and distress among them [112, 113]. Furthermore, they feared getting infected and additionally transmitting the disease to their family members and relatives [111]. Isolation and working with high-hazard departments and contact with contaminated individuals were considered as the most contributing reasons for trauma, fear, etc. [114] among HPs. A study showed that a substantial proportion (70.6%) of HPs reported stress during the COVID-19 pandemic [115]. Additionally, several studies demonstrated that the HPs experienced mental disturbances, anxiety, and nervousness to various degrees [102]. Our findings of a higher prevalence of depression, anxiety, stress, and insomnia are also consistent with previous findings.

Globally, COVID-19 has affected an enormous number of frontline HPs. In China, more than 3000 HPs were infected by COVID-19 until March 2020 [116]. A similar situation was also observed in previous outbreaks of SARS [117]. During the Middle East Respiratory Syndrome (MERS) outbreak, a Saudi report published that very nearly two-third of HPs felt in danger of getting infected with MERS-CoV and felt hazardous at work [118]. Several studies in Sierra Leone, Guinea, and Liberia observed nearly 6–8% of HPs infected by the Ebola virus [119]. Similarly, many early studies of COVID-19 among HPs found the reason for becoming infected by COVID-19 and as a result (Cavallo, Donoho, and Forman 2020), emerged fear and distress among HPs. As a result, the HPs are at an increased risk of contamination by COVID-19 and it would additionally worsen the current situation which could result in the lack of skilled health workers. Furthermore, HPs also feel vulnerable to getting contaminated and feared about the shortage of emergency medical equipment supplies and PPEs [120]. As a result, the HPs faced considerable stress, depression, obsession-compulsion, and feel unsafe to work as the infection rate increases [118]. Our findings are also consistent with the previous studies and found the higher pooled prevalence of depression, stress, anxiety, and insomnia.

Even though the present meta-studies found that mental issues are emergency health issues among the HPs as they are struggling with a highly infectious disease, most HPs are working in isolated wards without accepting sufficient support to improve their psychological health status. High-quality data on the mental health effects caused by the COVID-19 pandemic across vulnerable groups such as healthcare professionals is of massive importance [121]. Reviews and meta-analyses of this type are inevitable for supporting public health worldwide and to cut out the knowledge gap in the care of mental health disorders [122]. Therefore appropriate psychological support, encouragement, and motivational interventions, protective intervention, and staff support measures are urgently needed to address those issues. The funding bodies and governments can use these study results as a tool to ensure sustainable development in mental health by supporting the prioritization and allocation of funds for mental health.

## 5 Strengths

The main strength of this systematic review and meta-analysis was that we conducted the first comprehensive review on four major psychological morbidities namely depression, anxiety, stress, and insomnia during the COVID-19 pandemic. As per our knowledge, for the first time, we depicted the changes in the prevalence estimates of those psychological morbidities by three periods during the COVID-19 pandemic.

## 6 Limitations

Our studies have few limitations. First, all the studies we included, in the final analysis, were cross-sectional. Second, for the same test, different studies were considered by different assessment scales for screening health professionals. Third, among the included studies, some of the studies may consider the same population as the studies were conducted within the same country. Fourth, we may have missed including some articles in the final analysis as those were not published in English.

## 7 Recommendation

This study exhibited the need for further research and intervention on the mental health of healthcare workers. Especially, future investigations need to focus on identifying the responsible factors that are associated with the increase of the prevalence of depression, anxiety, stress, and insomnia. It is also crucial to observe the changes in those psychological morbidities from pre-COVID to COVID scenarios. Additionally, from the methodological point of view, to get more reliable estimates of psychological prevalence, researchers need to use the same assessment tools. We would like to recommend DASS-21 for assessing depression, anxiety, and stress because of its excellent consistency, convenience, and discriminative validity in results [123]. In some previous studies, this tool also showed adequate psychometric properties in the validation for healthcare workers and the general population [78, 124, 125]. As the most established and useful tool to quantify insomnia severity [126, 127], ISI should be selected.

## 8 Conclusions

To conclude, our systematic review and meta-analysis give a convenient and far-reaching amalgamation of the existing manifestation highlighting the high prevalence rates of depression, anxiety, stress, and insomnia among healthcare workers. The knowledge of the physical and mental health of the health care workers can help to implement interventions that are essential to enhance psychological resilience and strengthen the healthcare systems’ capacity.

## Declarations

### Author contribution statement

SM and MM conceived of the presented idea. SH and AM conducted the searches. They also completed the screening text, extraction, and analysis of the data with the input from SM. SH and SM wrote the first draft of the manuscript with input from MI and MM. MI, AM, and MM provided critical feedback. All authors discussed the results and contributed to the final manuscript.

### Funding statement

This research did not receive any specific grant from funding agencies in the public, commercial, or not-for-profit sectors.

### Data availability statement

Data included in article/supplementary material/referenced in article.

### Declaration of interests statement

The authors declare no conflict of interest.

### Additional information

No additional information is available for this paper.
